# LASTU: A psycholinguistic search tool for Finnish lexical stimuli

**DOI:** 10.3758/s13428-024-02347-x

**Published:** 2024-02-22

**Authors:** Sami Itkonen, Tuomo Häikiö, Seppo Vainio, Minna Lehtonen

**Affiliations:** 1 University of Turku, Turku, Finland; 2 University of Helsinki, Helsinki, Finland

**Keywords:** Finnish, Frequency, Lexical variable, Orthographic neighborhood, Psycholinguistics, Software, Stimulus search

## Abstract

*LASTU* is a tool for searching for Finnish language stimulus words for psycholinguistic studies. The tool allows the user to query a number of properties, including forms, lemmas, frequencies, and morphological features. It also includes two new measures for quantifying lemma and form ambiguity. The tool is written in Python and is available for Windows and macOS platforms. It is available at https://osf.io/j8v6b/. Included with the tool is a database based on a massive corpus of dependency-parsed Finnish language data crawled from the Internet (over 5 billion tokens). While *LASTU* has been developed for researchers working on the Finnish language, the openly available implementation can also be applied to other languages.

## Introduction

When researchers in psycholinguistics, speech-language pathology, or cognitive neuroscience compile stimuli for studies on language processing, it is usually essential to obtain information about lexical characteristics such as word frequencies, lengths, and orthographic properties. This has often been done in order to manipulate one lexical property (such as morphological structure or surface frequency) between stimulus conditions and to match the conditions for all the other relevant properties, or to investigate the contribution of different (sub)lexical properties on language processing in a multivariate design. The available tools for extracting this information from suitable corpora are varied and depend on the language of interest. Furthermore, their use may require programming skills and detailed knowledge of corpora which, for example, students of these disciplines may not possess. We present the software *LASTU*^1^ with an easy-to-approach user interface and which can be used to search for suitable words (forms or lemmas) based on specified (sub)lexical and morphological properties from a dependency-parsed corpus. It can also be used to extract such properties for ready-made word lists. While *LASTU* has been developed for researchers of Finnish, the program and its source code are openly available, and databases can also be created for other languages using the tools supplied in the source code.

Finnish is a morphologically rich language. With 15 cases and productive affixation, there are thousands of potential forms for each noun or verb (Koskenniemi et al., 2012), with derivation and compounding further increasing the complexity. However, although Finnish is typically called an agglutinative language, it also has fusional features (Dahl, 2008; Lehtinen, 2007). The morphological features cannot be completely predicted purely based on the surface form; thus we have a need for software and a database where the data has already been processed and linguistically analyzed.

The present software tool is to be considered a successor to the WordMill program (Laine & Virtanen, 1999). The original software was built in the 1990s and it is now only usable on computers running an old version of Windows. Despite being old and not openly available, WordMill has been actively used to this day (see, e.g., Perea et al., 2022; Schroeder et al., 2022 for most recent references), which indicates a clear need for this kind of a tool. Our new program is cross-platform and standalone, and is based on modern, but mature, software frameworks. Unlike its predecessor, it also provides a tool for building new databases for the program (in the GitHub source code repository).

Other, currently available tools for Finnish and their search possibilities do not satisfy the needs of researchers and students who are studying language processing. Most of the existing solutions use sentence-level data, while the fundamental purpose of our software is querying words or lemmas and their frequencies, not words or expressions in context. For example, a common solution for querying corpora is Corpus Workbench (CWB) (Evert & Hardie, 2011) that is designed for corpus analysis (e.g., concordances).

The main issue with tools like it is that one cannot query for words based on their properties and/or statistics (e.g., “give me all adjectives with relative frequency between 0.001 and 0.0001 and length between 6 and 10”). Other solutions such as using the Finnish Language Bank user interface or API^2^ have similar issues.

In addition to WordMill (1999), the current software has also been inspired by LexOPS (Taylor et al., 2020) for its feature set. Furthermore, the database and its building process took inspiration from the corpus of psycholinguistic descriptives of Finnish language assembled by Huovilainen (2018).

The source data for the database used by the program is a set of Universal Dependencies (UD) parsed files. Universal Dependencies (de Marneffe et al., 2021) is a framework for morphosyntactic annotations that are cross-linguistically consistent (e.g., standard word classes). UD-annotated databanks are openly available for more than 100 languages.^3^ For the universal morphological features supported by UD, please see de Marneffe et al. (2021, p. 263).

The main differences to the old program (i.e., WordMill) in terms of data are: (1) the database is modern and bigger (22.7 million vs. 5.5 billion tokens), (2) the frequencies and tags are based on dependency-parsed sentences (i.e., they separate words’ different grammatical functions in the sentences of the corpus) instead of context-free morphological parsing, and (3) in addition to being disambiguated based on the lemma, word surface form, and word class, the data is also disambiguated based on the “core” morphological features.

The features that were selected for Finnish^4^ are: case, number, and person; verb form (finiteness), mood, tense, and voice; clitics and derivations and the person and number for possessive suffix. However, the data are not disambiguated based on the word sense as this is not the kind of information that is obtained with dependency parsing. Table 1 shows an example for the word *silmäsi*, which is a form of the noun *silmä* (‘eye’).

The selection of the core features was done to achieve a balance between having relevant features and avoiding excessively long feature strings and combinatory explosion. The dependency parser is not completely reliable when tagging morphological features, which may result in noise. All selected features were to be searchable and disambiguated. Features provided by the parser that were dropped from the program included Degree^5^ (for adjectives) and Polarity (negative or positive) and extended features such as number or pronoun type and Style (e.g., colloquial). Features that do not exist in the Finnish language such as gender, definiteness, and aspect were obviously not also included.

**Table 1:** A subset of the features shown for the noun *silmä* (’eye’) with form *silmäsi*

lemma	lemmafreq	form	pos	frequency	feats	case	number
silmä	2108963	silmäsi	NOUN	13464	Case=Nom|Number=Plur	Nom	Plur
silmä	2108963	silmäsi	NOUN	4600	Case=Gen|Number=Sing	Gen	Sing
silmä	2108963	silmäsi	NOUN	1307	Case=Nom|Number=Sing	Nom	Sing

This form has three valid analyses: (1) plural nominative (‘your eyes’) (2) singular genitive (‘of your eye’), and (3) singular nominative (‘your eye’). The frequency column shows the frequency for the specific set of core morphological features (shown in the feats column)

## Goals

The goal was to develop a modern program to be used for searching for stimuli for psycholinguistic studies. To make the software easy to use, it needed to be usable without extensive technical knowledge. Furthermore, the users should not be expected to know programming languages or how to run open-source software in the source form.

### Features

We included all of the features that were available in the original WordMill program. First, lexical and sublexical features were included: lemma, surface form, average bigram, and initial/final trigram frequencies, word length, and class (i.e., part-of-speech), as well as a variety of morphological features. Furthermore, information whether a word was a compound or a proper noun (e.g., first name of a person) was also included. Second, the frequencies should not be case-sensitive as we wanted the program to provide “pure” frequency values. Thus, the word forms and lemmas needed to be stored in case-insensitive form. Third, it was considered useful to be able to search for words based on initial, final, or middle characters (e.g., words ending with *na*).

Searching from word lists was also deemed essential, as well as providing bigram and trigram frequency values for lists of non-words. Finally, all of the features needed to be searchable.

### Design choices

Developing software using modern tools can be challenging. The foundation that software is built on can be “a moving target”, undergoing rapid evolution. Recent examples of this include the development of JavaScript UI frameworks such as React and the ongoing revolution with large language models (LLM). In other words, choices regarding software architecture may have long-term consequences. Therefore, to have a stable foundation, the program should be built on technologies that are mature, yet have longevity, are well supported, and contribute to the maintainability of the software.

The programming language to be used deserved consideration. The first option was Python, which is the most popular language when it comes to language technology. In the field of psychology, however, R is often used. In the end, Python was chosen for two reasons: (1) the building of the database would require language technology components, which are readily available for Python and (2) the end users were not expected to be able to program themselves, which limited the potential usefulness of writing the software with R.

Another question was how the software would be used. To minimize complexity, the software should be standalone with no external dependencies, such as servers accessed through the net or database applications installed on the user’s computer. As the end-users were assumed to be non-technical, this ruled out the possibility of simply providing a Python script or a Jupyter Notebook^6^ as the end solution. The primary platform to build the application for was Windows, while macOS would be useful to be supported as well.

There were a number of choices for a GUI framework, including wxPython,^7^ Kivy^8^ and PyQt.^9^ WxPython was the oldest and most mature framework, while Kivy would be especially useful for building a mobile application. In practice, with wxPython, packaging the application to an executable turned out to be unnecessarily cumbersome. In the end, the PyQt framework was chosen for building the interface, as it was quite trouble-free and also well supported.

The database for the application needed to be embedded so that there was no need for a separate database application. The fundamental choice when considering a database is choosing between a SQL (row-based relational) and a document database (such as MongoDB). There are few standalone document databases, and their scalability is questionable, which made them unsuitable for our purposes. For an SQL database, picking SQLite3 was an easy choice: it is compact, cross-platform, and reliable (Gaffney et al., 2022). It needs to be noted, though, that the performance of an embedded database may not be as good as that of a standalone database server, which has an impact on the maximum possible size of our database.

## User interface

The application can be used in two modes: (1) searching the database based on a number of features (free search mode), and (2) using an input file to fetch entries from (wordlist mode). In the wordlist mode, the user can additionally generate N-gram frequency values for nonwords (i.e., strings that are not valid words in Finnish).

**Fig. 1:**
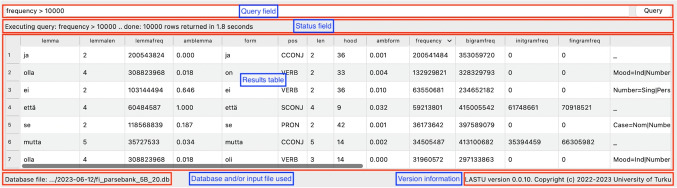
The screenshot shows the basic fields and tables in the *LASTU* user interface. At the top is the query box; queries can be executed with the Enter key or by pressing the Query button on the right. Below this field there is the status field, which shows whether the query is ongoing or finished, the number of results as well as the execution time. Most of the space is taken by the results table. The bottom row shows information about the current database and also the wordlist file if the application is in wordlist mode on the left side and version information on the right side. The *red boxes* as well as *blue boxes with text* have been added for demonstration purposes and do not appear in the actual program

The basic user interface is shown in Fig. 1, with query, status, database information and version information fields, and the results table. The results table contains information in four categories: lemmas, forms, frequencies, and features. The table is sortable by all columns. Numeric information that can have non-integer values (ambiguity percentages and relative frequencies) are shown with a precision of three decimals.

The application is multithreaded. Multiple windows and databases can be open at the same time. Some application features can be configured via a configuration file in the INI format.^10^ The results can be exported to clipboard or an Excel, CSV, or TSV file. Queries are logged to a history file; there is also a more general log file.

### Query language

The queries consist of query parts separated by the word and:

- PART
- PART and PART
- PART and PART and PART and ...

The query parts take the form key operator value (with some exceptions). All input is lowercased before feeding it to the database (that is, case = ine and case = Ine are considered equivalent). The underlying data, however, contain both lowercased properties (form, lemma) and TitleCased properties (any morphological properties that come from the UD-formatted source files).

The query grammar is simpler than that of the predecessor WordMill (Laine & Virtanen, 1999). For example, our queries do not support parentheses or *OR* queries. If the user needs to find words whose properties are in a specific set, the query operator *IN* must be used. For example, to find words that are in either inessive or genitive case, the query would be case in ine,gen whereas an SQL-like query would be something like (case = “ine” OR case = “gen”) (which is what the former expression is actually converted to). The goal was thus to prioritize user-friendliness while still keeping most of the power of more complex query engines.

**Table 2:** Example queries

query	explanation
form = autossa	Surface form
case = ine	Words in inessive case
lemma = voi and pos = noun	Nouns with the lemma voi

Table 2 shows a number of example queries. For a full list of supported query keys and operators, please see Appendix A.

## Features

### Word features

In *LASTU*, searches can be done on the basis of standard characteristics of the language, in this case Finnish. These basic word features include word class, whether a word is a compound or not, proper nouns, suffixes, and clitics, for instance. As noted above, it is also possible to search for words in which particular characters (e.g., *sa*) comprise the beginning or the end of the word, or where particular characters are included within the word. In the following sections, we will go over the included characteristics and their theoretical underpinnings.

### Basic characteristics

The database contains frequencies for both lemmas and forms of the words. Frequency effects are amongst the most robust word characteristic effects in visual word recognition (Whaley, 1978) and reading, e.g., (Inhoff & Rayner, 1986; Rayner & Raney, 1996), as well as spoken word recognition, e.g. (Dahan et al., 2001) and speech production, e.g., (Jescheniak & Levelt, 1994; Meschyan & Hernandez, 2002). Furthermore, both lemma and form frequencies have a unique effect on word recognition (Taft, 1979).

For the lemma frequencies, the database uses the combination of lemma and word class to define unique lemmas. For the form frequencies, the combination of lemma, word class, surface form, and feature string is used to define unique combinations. In other words, there is only one row in the database where the value for each of these columns is the same (see, for example, Table 1 where each of the feature strings for the two last rows is different). Raw and relative frequencies (value per 1 million tokens) are provided for both lemma and form. The relative frequency information is based on the actual data stored in the database. Thus, if the database has been pruned in any way (such as applying a minimum frequency), this is reflected in the both the raw and relative frequencies.

Moreover, word length, a variable typically extracted for stimulus words, is included in the database. It has been shown that the number of letters in a word correlates with its processing speed for children, neurologically healthy adults as well as participants with reading disorders; see Barton et al. (2014) for a review.

### Aggregated features

A number of features were calculated based on the basic frequencies of the whole database. First, the basic aggregated frequency measures are initial trigram, final trigram, and average bigram frequencies. Second, ambiguity percentages were calculated for lemmas and forms. Third, the orthographic neighborhood of word forms was calculated. In the following, we will go over some of the findings related to how these characteristics affect word processing, thus highlighting the need to include such properties in the tool.

Even though the findings concerning the effect of bigram frequency on visual word recognition are mixed and more recent studies often fail to find such effect (see Chetail, 2015 for a summary), there may be cases where bigram frequency has a role, such as reading aloud (Schmalz & Mulatti, 2017). Furthermore, in Finnish, Bertram and Hyönä (2007) demonstrated that the initial trigram frequency of the upcoming word had an effect of reading the currently fixated word. This is in line with the finding that the characteristics of the word beginning are extracted from the upcoming word even for children (Pagán et al., 2016).

Finally, while the final trigram frequency effects have been reported in the literature quite rarely; however, see e.g., Kuperman et al. (2009), they have been often matched during stimulus creation, (e.g., Häikiö et al. 2011; Pollatsek et al. 2000).

When a reader encounters a word that has more than one possible meaning, i.e., an ambiguous word, it slows down their reading (e.g., Rayner and Duffy, 1986). In fact, it seems that ambiguous words are represented in a different fashion in the mental lexicon than words with no ambiguity; see Eddington and Tokowicz (2015) for a review. As Finnish is a morphologically highly productive language, ambiguity can be found both at the form and lemma levels. Using ambiguous words as stimuli without paying attention to this characteristic might lead to serious confounds (see Sections Form ambiguity and Lemma ambiguity)

As for the orthographic neighbors (i.e., words that differ from another word by changing one letter), the term was introduced to the literature by Coltheart et al. (1977). There is a body of research showing that when a word has orthographic neighbors that are activated, it has an inhibitory effect on lexical access; see Perea and Rosa (2000) for a review. Because of this, various psycholinguistic tools and databases offer the neighborhood size as one of the variables, e.g., (Barca et al., 2002; Davis, 2005; Esmaeelpour et al., 2022; Witte et al., 2021).

#### N-gram frequencies

The initial trigram frequency (initrigramfreq) of a word form is the cumulative frequency of the first three letters of the word. Similar calculation is done for the final trigram frequency (fintrigramfreq). The minimum word length for both is four. The decision to have such minimum length was philosophical; for a three-letter word, it can be argued that the trigram that forms the whole word cannot be thought of being simultaneously the first and last three letters. These features are presented with both absolute and relative values; the latter values are calculated per 1 million tokens. For example, the relative initrigramfreq shows how many times out of million words a word begins with that particular trigram.

The bigram frequency of a surface form (bigramfreq) is the average cumulative frequency of the bigrams (i.e., two successive characters within the word).^11^ The bigram features are also presented with both absolute and relative values. When the bigram frequency is scaled instead of using database-dependent raw values, it is usually scaled per million tokens, e.g., (Conrad et al., 2009; New et al., 2001; Oganian et al., 2016). Thus, the scaling factor for the relative bigram frequency in this database is 1 million.

#### Form ambiguity

Form ambiguity (ambform) is the weighted probability that the lemma and word class for a form are likely to be something else than the specified lemma and word class. For example, the form *voi* is the base form for the noun *voi* (’butter’); it can also be an interjection (’oh’). However, the form will far more likely refer to the verb *voida* (’to be able to’), leading to a high ambform probability for the noun *voi*. See Table 3 as an example of this. The ambform is presented as a decimal fraction where 0 denotes no ambiguity whatsoever and 1 denotes that all of the occurrences of said form can be classified to another lemma/word class combination.

**Table 3:** A subset of the data for the form *voi* from the Finnish Internet Parsebank (Luotolahti et al., 2015) data

lemma	lemmafreq	pos	frequency	ambform	amblemma	feats
voida	34219997	VERB	7810951	0.050	0.512	Number=Sing|Person=3|Tense=Pres|VerbForm=Fin
voida	34219997	VERB	4196751	0.050	0.512	Number=Sing|Person=0|Tense=Pres|VerbForm=Fin
voida	34219997	VERB	3655767	0.050	0.512	Tense=Pres|VerbForm=Fin
voi	752892	INTJ	750292	0.954	1.000	_
voi	168690	NOUN	52296	0.997	0.913	Case=Nom|Number=Sing

This word will overwhelmingly likely be a form of the verb *voida* (‘to be able to’), reflected by the low form ambiguity percentage (ambform) for the verb and high percentage for the other choices. For the interjection interpretation, the lemma ambiguity percentage (amblemma) is 100% (i.e., 1.000) as an interjection cannot be inflected in Finnish

#### Lemma ambiguity

In WordMill (1999), lexeme’s base is determined by the base form that the morphological parser (FINTWOL by Lingsoft two-lc) has given. If a lexeme has more than one base, it is then marked as ambiguous. The lemma ambiguity for any given base form is then the percentage of lexemes contributing to said base form that have been marked ambiguous. The parsing method is context-insensitive.

In *LASTU*, lemma ambiguity (amblemma) is the weighted probability that a surface form belonging to the lemma/word class combination is ambiguous. In principle, a surface form is unambiguous if it is only used with this lemma. In practice, however, some allowances need be made due to noisiness of the data. Thus, a form is considered unambiguous if its ambform percentage is below 1% (i.e., below 0.01). See Tables 3 and 4 for examples. Similarly to form ambiguity, amblemma is presented as a decimal fraction.

**Table 4:** The most frequent forms for the noun *voi* (’butter’) from the Finnish Internet Parsebank data

form	frequency	ambform	feats
voita	64719	0.504	Case=Par|Number=Sing
voi	52296	0.997	Case=Nom|Number=Sing
voin	17869	0.984	Case=Gen|Number=Sing
voissa	13453	0.002	Case=Ine|Number=Sing
voilla	5347	0.026	Case=Ade|Number=Sing
voista	3241	0.033	Case=Ela|Number=Sing

Only some word forms – in terms of number and percentage – are unambiguously a form of this lemma. The ambform percentage of *voissa* is below 1% (i.e., below 0.010), thus it will be considered “unambiguous” for the purposes of the lemma ambiguity (amblemma) calculation

#### Orthographic neighborhood

The orthographic neighborhood (hood) is considered as the set of forms in the database where the Hamming distance from the form is 1, that is, substitution of one letter (following the example of Taylor et al., 2020). For calculating the neighborhood (when building the database), the system uses a spelling dictionary with the symspell^12^ Python module and the uralicNLP^13^ morphological analyzer.

As the database contains a lot of noise, the forms that contribute to the orthographic neighborhood size were filtered based on the surface frequency and the morphological analyzer with the following criteria for inclusion and exclusion: (1) if the frequency was high enough (autofreq), the form was included automatically, 2) if the frequency was too low (below minfreq), the form was excluded, and (3) for the frequencies in-between, the morphological analyzer was used to determine if the form was a valid Finnish word in order to be included. The values for autofreq and minfreq were set to 10,000 and 100, respectively. These values were derived experimentally during the testing process and have not been adjusted for databases of other sizes.

### Wordlists

As the user may need to fetch characteristics for a list of words when, for example, conducting a corpus analysis, both surface and lemma wordlist searches were included. In case the user needs to extract information for nonwords for a lexical decision task, for example, the wordlist may also comprise of nonwords. The results provided by the wordlist mode are further filterable and searchable by the same features as in the free search mode.

## Database building

The initial data for developing the application came from Finnish language books of Project Gutenberg,^14^ which were parsed to Universal Dependencies (Nivre et al., 2020) format with the Turku Neural Parser (Kanerva et al., 2018). The Gutenberg dataset contained nearly 2000 books with 65 million words in 6 million sentences. For comparison, the database for the original WordMill software was based on a corpus of 22.7 million words.

The desired end result was one large corpus. The target was to have high accuracy for syntactically and grammatically correct Finnish; correctly tagging typos was not important. All data were to be lower-cased.

Some consideration was given to how the source data were to be parsed. Rule-based parsers would have consistent tagging, while being sensitive to correct grammar. Conversely, state-of-the-art neural parsers have good performance with unknown forms and have higher overall accuracy, yet there is no guarantee that the same kinds of sentences are consistently tagged the same way. As a big corpus was desired, the data needed to already be available in parsed form. Since modern dependency treebanks are parsed with neural parsers, this meant that the choice regarding the parser had already been made for us.

The practical end result is that the data is noisy: it will have analyses that are incorrect in many ways (wrong word class, wrong noun case, etc.). The expectation, however, is that such errors will generally be of low frequency.

### Aggregated features

Certain verbs can be used as regular verbs (*VERB*) or auxiliary verbs (*AUX*). To distinguish between cases where one is interested in verbs in general or specifically in auxiliary verbs, the column posx was created in the database to combine regular and auxiliary verbs under a single tag (*VERB*). This column is used for most queries. When specifically querying auxiliary verbs (e.g., pos = aux or pos != aux or pos IN aux), the original column pos is used.

This method created a lot of extra work for the queries and necessitated the creation of additional database indexes for searching, which also had an impact on the database size.

### Final database building

The final dataset(s) were built from the Finnish Internet Parsebank (Luotolahti et al., 2015) with 5.5 billion tokens and over 500 million sentences in 530 files.

The data were pre-filtered before importing it to the SQLite3 database with the main objective of dropping the most obviously unnecessary tokens. The first pruning measure was filtering out tokens (lemmas/forms) that included letters that do not exist in the Finnish language (certain punctuation characters were allowed). The token also had to begin with an alphanumeric character and contain at least one actual letter. The minimum length of the token was two characters as Finnish does not have any one-character words.

For performance purposes, the database was built from multiple parts: a database file was created for each source file. Six database files were combined from these files (with up to 100 source files per database), and the final database was combined from these six. Combining the database files was done with the SQLite UPSERT^15^ method: When a row exists in both databases, the frequencies are summed up. This method might not be the most efficient one for combining the rows, but it is simple and straight-forward.

The final database(s) were pruned based on a minimum frequency (of lemma/form/word class/feature combination). The frequency was chosen so that the program would still be usable with normal laptops (that is, the execution time of the most challenging queries would be reasonably short). The minimum frequency for the final two databases was 10 and 20 (for comparison, the data for psycholinguistic descriptives (Huovilainen, 2018) with 2.5 billion tokens was filtered based on minimum frequency of 0.01 tokens per million, which amounted to a minimum surface frequency of 27). Tokens with word classes *PUNCT* (punctuation), *SYM* (symbol) and *X* (no valid word class found) were also filtered out.

As mentioned before, the data is noisy, as it was crawled from the Internet. The noise includes words that are excessively long, which should be filtered out. For example, the unfiltered database contained 143 words that occur at least ten times and are longer than 100 characters. An examination revealed them to be artifacts of the method that was used to parse the source HTML documents, with the parser mistaking these strings as part of the textual “meat” of the document. For example, these strings may represent concatenated table headers or be part of the JavaScript code accompanying the HTML document.

The question then became: What should the cutoff value be for length? Finnish words theoretically can have almost unlimited length due to productive compounding and derivation. Our database does contain the word *epäjärjestelmällistyttämättömyydelläänsäkäänköhän* with a length of 49 characters - slightly shorter than its often-quoted variant^16^ – but this is clearly meta usage (as it is used as an example of a long word in Finnish). Excluding frivolous and made-up examples, the longest actually used words generally max out between 30 and 40 characters.^17^ The final cutoff length was a very conservative 100 characters.

Indexes and calculated features were added to the database only after pruning. Adding the indexes increased the database file size 6–7 times.

## Demonstrations

In this section, we show that *LASTU* works effectively in searching and narrowing down specific stimuli that have been used in psycholinguistic studies of Finnish. We focused on one published study (Laine et al., 1999) which included quite specific inflected and pseudoinflected Finnish words as stimuli and which reported all the target words explicitly in the article. We attempted to mimic the search process of one experiment from this study (Experiment 1) with *LASTU*. We used the *fi_parsebank_5B_20* database (built from Finnish Internet Parsebank (Luotolahti et al., 2015) with minimum surface frequency of 20) for the searches.

Note that stimulus selection is usually not a process that can be fully automatized even with available tools, but researcher evaluation is needed at several steps, and matching of word groups typically involves trial and error. Also, there is usually not only one possible solution for stimulus lists, but the number of potential items and item combinations may be large. The searches may thus produce long item lists if the options are plenty. Similarly, the words obtained by *LASTU* will require researcher consideration and selection of potential stimuli from a larger set before reaching the final stimulus lists.

Laine et al. (1999) studied recognition of morphologically complex words in Finnish (e.g., *talo+sta* = ‘from a house’) and, among other things, whether this happens via automatically stripping off the affix (e.g., the elative ending “-sta”) from the complex words before accessing the word stem (*talo* = ‘house’). To this end, in Experiment 1 they studied whether the presence of a pseudosuffix in an uninflected word hinders word recognition, which would suggest that morpheme-based processing is activated by the mere presence of a suffix-looking word ending. For a visual lexical decision experiment, they collected (A) nouns including pseudosuffixes, i.e., uninflected nouns ending in letters that are also suffixes in the language (e.g., ending “-sta” in *kiista* (’dispute’); cf. word “bia+s” in English with a pseudosuffix “-s”), together with control conditions: (B) truly case-inflected Finnish nouns (e.g., *ura+sta* = ’from a career’), as well as (C) nominative singular nouns without pseudoinflections (e.g., *anoppi* = ’mother-in-law’). The original stimulus lists and the present search results can be found in the Supplementary Materials (Tables A-C) (in the OSF repository).

Additionally, pseudowords that were either non-morphemic or carrying inflectional suffixes (Condition D) were included for the lexical decision task. However, these items were not reported in the article, and we therefore disregard them in the present demonstration. It should nevertheless be noted that it is possible to render bigram and trigram frequencies also for pseudowords for matching purposes by running the lists via *LASTU*’s input command.

### Condition A

We first conducted the search for the pseudoaffixed words with the following command:

pos in NOUN,NUM and end in sta,na,a and case = Nom and number = Sing and len> 5 and len< 9 and rellemmafreq> 1 and relfrequency> 0.1 and not compound and posspers not in 1, 2, 3 and clitic not in Kin, Han and derivation != Ja and ambform< 0.95

This search allowed us to find word forms that included a pseudosuffix while being monomorphemic words. While the focus was on nouns, one numeral word was included in the original set of Laine and Virtanen (1999), and we thus allowed the search to include numerals as well (pos in NOUN,NUM). As for the pseudosuffixes, the words needed to end in a specific letter combination that is also a suffix in the language while not being inflected words but of nominative case (case = Nom). The selected pseudosuffixes included elative (“-sta”), essive (“-na”), and partitive (“-a”) endings (end in sta,na,a). Only singular words were included (number = Sing), and the length of the words was restricted to 6–8 letters (len> 5 and len< 9). Very low frequency words were excluded, with an arbitrary boundary of 1 per million for lemma frequency (rellemmafreq> 1) and 0.1 per million (relfrequency> 0.1) for word form frequency. Compound words were excluded to avoid additional morphological complexity (not compound). We also excluded other potential morphological endings such as possessive suffixes and clitics (posspers not in 1, 2, 3 and clitic not in Kin, Han). As the initial result produced several items with the productive derivational ending “-ja”, we excluded those with the command (derivation != Ja). The search also found some incorrect items due to corpus parsing issues, and we therefore added a command (ambform< 0.95) which effectively omitted most of such items.

See Table A in the Supplementary Materials for the search results. All the original target items were found by this search strategy. The search produced altogether 568 rows of which the overwhelming majority could be seen to be potentially usable items for the purpose of the experiment. The rest were mainly compound words not picked up by the parser, foreign words, or colloquial words which are not tagged in the corpus, and a few incorrect parses in the corpus. The relatively large number of rows found reflects the commonness of the final letter “-a” in the Finnish language; when this pseudosuffix was excluded, the number of rows dropped to 73 for endings “-sta” and “-na” together.

### Condition B

Second, we conducted a search for case-inflected Finnish control nouns with the same suffixes that had been used for pseudosuffixes for Condition A. For this purpose, we reran the same search, attempting to find words with the same word endings but only when they were included as actual suffixes. Therefore, we replaced the case from nominative to either elative, essive, or partitive (case in Ela,Ess,Par). The search command was as follows:

pos in NOUN and end in sta,na,a and case in Ela,Ess,Par and number = Sing and len> 5 and len< 9 and rellemmafreq> 1 and relfrequency> 0.1 and not compound and posspers not in 1,2,3 and clitic not in Kin,Han and derivation != Ja and ambform< 0.95

See Table B in the Supplementary Materials for the search results. This search produced a result with 4007 rows, which included all the original target words. In addition to the original items, the overwhelming majority of the other items produced were also potentially usable for this purpose. Words that might have been nonideal for the present experiment included colloquial words or foreign words present in the corpus material. Such words cannot be filtered out automatically by *LASTU*.

### Condition C

Third, we searched for monomorphemic control nouns without pseudoinflections with *LASTU*. Again, we kept the search string as similar as possible to the other searches but replaced the essive, elative, and partitive case for nominative and excluded potential pseudosuffixes (not only the ones used in Condition A but also other Finnish nominal case endings which include the final letters “-a”, “-ä”, “-n” as well as “-lle”, and “-ksi”; note that the first three of these effectively exclude also the other case endings, such as the elative “-sta”). We also filtered out the results for unusable entries ending in a hyphen (“-”). The search command was as follows:

pos in NOUN and end not in a,ä,n,lle,ksi,- and case = Nom and number = Sing and len> 5 and len< 9 and rellemmafreq> 1 and relfrequency> 0.1 and not compound and derivation != Ja and posspers not in 1,2,3 and clitic not in Kin,Han and ambform< 0.95

See Table C in the Supplementary Materials for the search results. This search produced 3176 rows, including all but one of the original stimulus words: there was one item *hevonen* (’horse’) in the stimulus set that technically includes a pseudosuffix (“-n”, a genitive marker). Again, a clear majority of the words could have been usable for the list, apart from mainly foreign words and colloquial words.

## Limitations

Finnish is a so-called orthographically transparent language (Georgiou et al., 2012), or in layman’s terms “spoken as it is written”, with very few exceptions. Thus, information regarding pronunciation and other phonological variables are generally not relevant for our needs.

The orthographic neighborhood calculation uses the uralicNLP module for assisting in determining the validity of neighboring words in Finnish. The neighborhood values will be less accurate for other languages.

Morphological family size was not included as the feature had not been present in the original Wordmill software. There are also limitations when considering its inclusion in the database. Firstly, while the dependency-parsed source material may contain information about derivations, there is no information about what the base word may be, e.g., it does not know that *kirjasto* (‘library’) is derived from *‘kirja’* (book). Compound information, however, would be available.

Secondly, family size calculations are limited by the lack of linguistic consensus related to productivity of Finnish derivative affixes, i.e., what are the productive, semi-productive and non-productive affixes. Thus, the developers of automatic parsers have had to make tough decisions as to which affixes are included. Naturally, this decision can have a huge impact on the family size calculations. This potential inaccuracy would mean that the family sizes should be double-checked manually to reach an acceptable level of reliability, which is virtually impossible due to the size of the corpus.

When the result set is large (with the default maximum value for rows being 10,000), the user interface can be slow when displaying the initial results or sorting them, depending on the computing resources of the user’s computer. This slowness is inherent in the implementation of PyQt and cannot be easily fixed. Users experiencing this issue are recommended to lower the value of the maximum number of rows to fetch in the .ini configuration file.

The query language is SQL-like, but it only supports the AND operator for combining query parts. More importantly, the queries do not support the OR operator. Based on the examples from the original WordMill program, most use cases with this operator related to searches within a single property and can be handled with the IN operator (e.g., end in ssa,ssä, i.e., the surface form ends in ssa or ssä, that is, inessive case). An example of a query that cannot be handled with current implementation is len< 10 OR len> 20, that is, surface form is either shorter than ten characters or longer than 20 characters.

### Database noise

As noted earlier, the database contains noise due to the fact it has been parsed by a neural parser and has not been curated in terms of whether word classes or morphological features have been correctly tagged. It should be noted, however, that these are not limitations in our software, just the accompanying data and we have taken steps to alleviate these issues (to an extent) with the ambiguity percentages. Table 5 shows examples for the word form *voit*.

**Table 5:** Examples of noise in the database due to parsing for the surface form *voit*, aggregated over all combinations of core features for the lemma/form/word class triplet

lemma	word class	frequency
*voida*	VERB	3075975
voida	NOUN	7425
voit	NOUN	4288
*voi*	NOUN	2700
voida	PRON	1711
voit	PROPN	1218
voida	ADV	1045
voit	VERB	986
voit	ADV	786
voida	ADJ	623

The only valid lemmas for the form are the verb *voida* (‘to be able to’) and the noun *voi* (‘butter’) (shown in italics)

These issues are also not unique to our dataset; the original WordMill (Laine & Virtanen, 1999) software also had noise in the database. Moreover, while the neural parser used for parsing the original text data is probabilistic, the original WordMill used a rule-based system for getting morphological analyses. These morphological analyses could roughly be expected to be correct (i.e., no noise), but – as with all rule-based systems in general – it was sensitive to out-of-vocabulary issues (cannot recognize new words, colloquialisms, etc.). It also only knew the aggregate frequency of a surface form (e.g., it did not know about the relative frequencies of verbs versus nouns for the word *tuli*, which denotes either verb-form ’came’ or noun ’fire’).

Modern rule-based parsers might have been more suitable for the applications that our data will be used with. However, rule-based parsers have issues with noise as well and the Turku neural parser (Kanerva et al., 2018) that was used to produce the Finnish Internet Parsebank (Luotolahti et al., 2015) that we used as our source was the most accurate parser when it was released.

Regarding similar datasets, the corpus of psycholinguistic descriptives (Huovilainen, 2018) is also based on equally noisy data, but it is not as sensitive to inaccuracies, as it does not include morphological features, just form, lemma and word class. We are not aware of any applications or datasets that also include morphological information the way ours does.

Our dataset is no worse than its predecessors if morphological features are not relevant for the study (i.e., only lemmas, surface forms, and/or word classes are used). In other words, the issues in (Finnish) dependency-parsed data have always existed; it is our way of using the data that have brought them to the spotlight. When one drills down to a very detailed level, the issues start to manifest more clearly.

One should also distinguish between inaccuracies in discrete variables (word class, case) and those in numeric/continuous values (frequencies). Whether or not the noise will hamper a specific study largely depends on the study.

Curation is a challenging issue, to be sure. However, with large amounts of data, large-scale manual curation of the data is simply infeasible and, as a result, no completely reliable databases exist for Finnish.

### Extending to other languages

The set of supported morphological features is auto-detected from the database schema. When building the database, the feature set is taken from the database schema feature definition file features.sql. The default values are tuned for Finnish. For example: Finnish is a non-gendered language, and it does not have a grammatically marked aspect. Thus, the UD features Gender and Aspect are missing. To build a database for languages other than Finnish, one needs to create an alternate database schema file or uncomment the relevant lines in the database schema file, should it already exist. The user can then enable the alternate schema by using the *language* command line parameter for the builder script (the language code is stored in the metadata table in the database, so further helper scripts do not have to invoke the parameter explicitly).

The database building procedure filters out tokens that are not expected to be words in the given language. The token must start with a letter or a number and may contain some punctuation characters in the middle (such as a hyphen for Finnish compound words). The current method of filtering is based on regular expressions that work for a small number of languages, including Finnish, Swedish, Spanish, and Portuguese. To support other languages, the regular expressions should be updated to allow tokens that are legal in “any” natural language.

The current orthographic neighborhood calculation also utilizes a morphological analyzer for Finnish. A different morphological analyzer would be required for other languages to make the neighborhood values equivalent.

Our application may be more easily applied for languages that are also relatively transparent orthographically. Examples for such languages can be found in (Van den Bosch & Daelemans, 1994; Marjou, 2021) and in Wikipedia.^18^

For languages with frequent use of accent marks (such as Portuguese), searching without accent marks might be useful. While grammatically incorrect, in (very) informal communication, accent marks may be omitted. As corpora are usually built from Internet-crawled data, it might be useful for the application to treat the unaccented forms equivalently to their correct forms. This might require significant changes to the database underlying our software.^19^ For the Portuguese language, the issue might be compounded by the recent orthographic reform (Schmitz, 1998), whose main purpose was to unify the spelling of Brazilian and European Portuguese.

## Future considerations

For future databases, other word class tags could also be combined the same way as VERB and AUX were. Suitable candidates could be at least combining ADP (adposition) with ADV (adverb).

The process for building the application includes a phase for running tests against a small test database generated from the Finnish Gutenberg data, with the tests checking that the queries return expected results. Only a small portion of the potential features are tested, however. The test coverage should be increased in the future for increased software quality.

Finnish syllable features are not included in our database, but they are included in the Finnish psycholinguistic descriptives data set by Huovilainen (2018). This is a feature that could be added to our software as well.

The maximum supported database size is in practice set by the performance (i.e., execution time) of the slowest search operations. These generally involve matching a string to the form property. SQLite3 can use indexes when searching from the beginning of the string (e.g., form starts with *au*), but not in the middle (i.e., not matching to string *auto* when form should contain the string *ut*). This means that searching from the middle of the string is relatively slow compared to searching from the beginning of the string.

For SQLite3, a potential way to improve search performance is to use full text search.^20^ Other possibilities include using a different embedded database engine such as DuckDB^21^ or storing the database in PyArrow^22^ format and using PolaRS^23^ to query it. This might allow us to use plain CSV files as the database and do away with the complicated query architecture. If a high-performance solution for matching text would be found, it would also be possible to do away with separate features table(s) and match the feature string directly.

## Conclusion

In this work, we introduced *LASTU*, which is a tool for searching for stimulus words for psycholinguistic research. It has primarily been developed for Finnish, but it can also be applied to other languages. Our application is especially suitable for morphologically complex and orthographically transparent languages.

The program is built with mature, proven, and modern technologies. It has no external dependencies (web servers, databases) and can be used on any relatively recent laptop on Windows or macOS. The open-source version can also be used in any platform that supports Python. The software includes various statistical measures for words such as raw and relative frequencies and orthographic neighborhood as well as two new measures for lemma and form ambiguity.

Along with the software, we also provide a database for Finnish words based on a large dependency-parsed data set for Finnish concurrently with the software. Databases can be built for other languages as well using the tools provided in the source code.

## Appendix A: Query grammar

There are three types of query keys: string, numeric and Boolean. Tables 6, 7 and 8 describe the query keys and operators for string properties (for a Finnish language database), while Tables 9 and 10 describe them for numeric properties.

**Table 6:** Query keys for string properties

key	explanation
lemma	Lemma
form	Surface form
pos	Word class
number	Number
person	Person
posspers	Possessive suffix person
possnum	Possessive suffix number
mood	Verb mood
tense	Verb tense
verbform	Verb form
nouncase	Case
derivation	Derivation
clitic	Clitic

Noun case also has the shortcut case

**Table 7:** Operators for querying string properties

operator	explanation	example
=	Equal	form = kuusi
!=	Unequal	pos != noun
in	In a set	case in nom,gen
not in	Not in a set	case not in nom,gen
like	SQL LIKE operator	form like auto%

**Table 8:** Additional query keys for the surface form

key	explanation	example
start	Form starts with	start = au
middle	Form contains	middle in ta, sa
end	Form ends with	end not in ssa, ssä

The operators are the same as for strings except for LIKE, which is not supported. The middle query key matches if there is a match when the first and last characters are chopped off. It may also match in the beginning or the end. For example middle = na will match *banana*

**Table 9:** Query keys for numeric properties

key	explanation
lemmafrequency	Lemma frequency
frequency	Surface form frequency
lemmalen	Lemma length
len	Surface form length
hood	Orthographic neighborhood
amblemma	Lemma ambiguity percentage
ambform	Surface form ambiguity percentage
initgramfreq	Initial trigram frequency
fingramfreq	Final trigram frequency
bigramfreq	Bigram frequency

All frequency queries have the relative frequency equivalent, with the query key prefixed with the string rel. Lemma and surface form frequency queries also allow the shorter form freq and lemmafreq

**Table 10:** Operators for querying numeric properties

operator	explanation	example
=	Equal	hood = 10
!=	Unequal	amblemma != 0
>	Greater than	relfreq> 10
>=	Greater than or equal	len>= 10
<	Lower than	ambform< 0.1
<=	Lower than or equal	lemmalen<= 6

The wordlist mode has an additional query key top: top = 2 shows the top 2 results for a given lemma/word class/surface form combination.

There is only one Boolean query key: compound. It allows the user to query whether the lemma is a compound word or not.

## Appendix B: Sample queries from LASTU and WordMill

**Table 11:** Sample queries. Some of the WordMill examples are from the manual (Laine & Virtanen, 1999, p.17)

LASTU query explanation	WordMill query
form = tulen	word="tulen"
Word is *tulen*.	
start = epä	initial="epä"
Word starts with *epä*.	
middle != ng	middle<>"ng"
The sequence *ng* does not appear in the middle.	
It may still start or end with it.	
len>= 10 and len<= 20	len>=10 and len<=20
Word length is between 10 and 20 characters.	
len>= 10 and pos != VERB	len>=10 and not v
Non-verbs that are at least 10 characters long.	
not compound	not bndry
Not a compound word.	

## Footnotes

1 The name *LASTU* stands for *Lexical Application for STimulus Unearthing* through an invented Finnish metaphor *yksi sana on kuin lastu laineella* (*one word is like a chip on a wave*). The word *lastu* means *chip* (as in a *wood chip*).

2 https://korp.csc.fi

3 https://universaldependencies.org/

4 https://universaldependencies.org/fi/

5 All UD features are described in TitleCase.

6 https://jupyter.org

7 https://www.wxpython.org

8 https://kivy.org

9 https://doc.qt.io/qtforpython-6/

10 https://en.wikipedia.org/wiki/INI_file

11 Note that this definition is different from the one used in the language technology field, where a bigram is two successive words.

12 https://github.com/mammothb/symspellpy

13 https://github.com/mikahama/uralicNLP

14 https://www.gutenberg.org

15 https://www.sqlite.org/lang_UPSERT.html

16 https://en.wikipedia.org/wiki/Longest_words#Finnish

17 https://www.kielikello.fi/-/pisin-sana

18 https://en.wikipedia.org/wiki/Phonemic_orthography

19 However, the prevalence of such informal language is unknown. If the frequency of such usage is very low, it would make the necessity of the feature moot.

20 https://www.sqlite.org/fts5.html

21 https://duckdb.org/why_duckdb

22 https://arrow.apache.org

23 https://pola-rs.github.io/polars-book/

## Open Practices

Databases, documentation, supplementary materials and installable packages are available at: https://osf.io/j8v6b/.

The source code for program and for building databases for it is available under the MIT license at: https://github.com/dustedmtl/lastu.
